# Association between intellectual disability and autism spectrum disorder with kidney failure

**DOI:** 10.1007/s00467-026-07177-x

**Published:** 2026-02-01

**Authors:** Hye Yeon Koo, In Young Cho, Yong-Moon Mark Park, Kyung Mee Kim, Chung Eun Lee, Kyungdo Han

**Affiliations:** 1 Department of Family Medicine, Seoul National University Bundang Hospital, Seongnam, Republic of Korea; 2 Department of Family Medicine, Seoul National University College of Medicine, Seoul, Republic of Korea; 3 Department of Family Medicine and Supportive Care Center, Samsung Medical Center, Sungkyunkwan University School of Medicine, 81 Irwon-Ro, Gangnam-Gu, Seoul, 06351 Republic of Korea; 4 Department of Epidemiology, Fay W. Boozman College of Public Health, University of Arkansas for Medical Sciences, Little Rock, AR USA; 5 Cancer Prevention and Population Sciences Research Program, Winthrop P. Rockefeller Cancer Institute, University of Arkansas for Medical Sciences, Little Rock, AR USA; 6 Department of Social Welfare, Soongsil University, Seoul, Republic of Korea; 7 Department of Child Psychology and Education, Sungkyunkwan University, Seoul, South Korea; 8 Department of Statistics and Actuarial Science, Soongsil University, 369 Sangdo-Ro, Dongjak-Gu, Seoul, 06978 Republic of Korea

**Keywords:** Intellectual disability, Autism spectrum disorder, Developmental disabilities, Chronic kidney failure, Public health

## Abstract

**Background:**

Evidence regarding the risk of incident kidney failure based on developmental disabilities remains scarce. Accordingly, we examined the risk of kidney failure in individuals with intellectual disability (ID) and autism spectrum disorder (ASD).

**Methods:**

This retrospective cohort study used data from the National Disability Registry of Korea and identified all individuals registered with ID/ASD between 2004 and 2020. In total, 155,729 individuals with ID and 22,385 with ASD and their age- and sex-matched controls (*n* = 467,187, and 67,155, respectively) were included and followed up until December 2023. Cox proportional hazard analyses were performed to estimate hazard ratios (HRs) and 95% confidence intervals (CIs) for the risk of kidney failure in individuals with ID/ASD compared with controls.

**Results:**

A total of 1,058 kidney failure events were identified in the ID cohort (687 ID and 371 controls) and 20 events in the ASD cohort (16 ASD and 4 controls). Individuals with developmental disabilities had a higher kidney failure risk than controls, with adjusted HRs (95% CIs) of 5.50 (4.75–6.36) for ID and 8.62 (2.68–27.74) for ASD. In subgroup analyses, both ID and ASD were associated with an increased risk of kidney failure in most subgroups. Age, sex, income level, and comorbidities showed a significant interaction with ID in relation to kidney failure risk (all *p* < 0.01), whereas no significant interaction was observed between baseline characteristics and ASD.

**Conclusions:**

Individuals with ID and ASD had an increased risk of incident kidney failure. Tailored preventive strategies against kidney failure are warranted for this population.

**Graphical abstract:**

**Figure:**
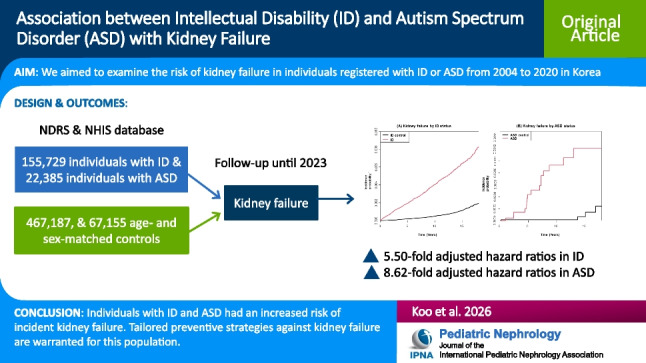
A higher resolution version of the Graphical abstract is available as Supplementary information

**Supplementary Information:**

The online version contains supplementary material available at 10.1007/s00467-026-07177-x.

## Introduction

Chronic kidney failure (hereafter referred to as kidney failure) represents a major global health burden, with the disability-adjusted life years and number of deaths attributable to kidney failure increasing steadily in recent decades [1]. Given the health risks and economic burden associated with kidney failure [1, 2], identifying high-risk groups for kidney failure and implementing rigorous surveillance are crucial for public health management.

Meanwhile, the increased burden of comorbidities in individuals with developmental disabilities has been reported and has attracted considerable research attention [3, 4]. Specifically, individuals with developmental disabilities demonstrate a higher prevalence of cardiometabolic disorders—including hypertension, diabetes mellitus, and obesity—compared with the general population [3–5]. Because these conditions are established risk factors for chronic kidney disease (CKD), at its most advanced stage kidney failure [6], it is plausible that the risk of kidney failure may be elevated in populations with developmental disabilities. However, only a few studies have explored the prevalence of CKD in individuals with developmental disabilities, reporting inconsistent results [3, 7]. Longitudinal evidence of the risk of incident CKD or kidney failure remains scarce. Given the recognized high disease burden and mortality risk among individuals with developmental disabilities [3, 4, 8, 9], if the kidney failure risk is also increased in this population, it is essential to establish appropriate screening and intervention strategies, making the identification of kidney failure risk in this group imperative.

In Korea, intellectual disability (ID) and autism spectrum disorder (ASD) are legally defined as developmental disabilities. Both are early-onset developmental disorders that are vulnerable to health disparities and are eligible for welfare benefits after registry in the National Disability Registration System (NDRS) [10]. The NDRS comprises information on all disabilities registered nationwide [11], enabling the analysis of data for nearly all individuals registered with these developmental disabilities. Therefore, we aimed to investigate the risk of incident kidney failure in individuals with developmental disabilities using a population-based nationwide Korean dataset. The objective of our study was to examine the kidney failure risk in individuals registered with ID or ASD from 2004 to 2020 in Korea compared with age- and sex-matched controls.

## Methods

### Data sources

We used data from the Korean National Health Insurance Service (NHIS) database, which is linked to the NDRS. The NHIS is the only insurer managed by the government and provides universal health coverage to 97% of the Korean population, with the remaining 3% in the lowest bracket covered by the Medical Aid program [12]. Hence, the NHIS is a population-based database that includes an eligibility database (age, income level, and residence) and health claims data (diagnostic codes and prescriptions). This study was approved by the Institutional Review Board of Soongsil University (IRB No. SSU-202312-HR-502-1). The need for informed consent was waived because a de-identified dataset was employed.

The NDRS was established in 1989 to provide welfare benefits according to disability type and severity [11]. To register in the NDRS, individuals must submit validated documentation, including diagnostic assessments by a specialist physician, to the National Pension Service following specific criteria for each disability type. The NDRS identifies 15 distinct disability categories based on the Korean Welfare Act for the Disabled. The NDRS database linked to the NHIS only provides information on the primary disability type, while data regarding multiple or secondary disability types are unavailable.

### Study population

We initially identified individuals registered with an ID (*n* = 157,821) or with ASD (*n* = 22,423) between 2004 and 2020. The index date was defined as January 1 of the year following the disability registration year, since the NDRS provides only the year of disability registration but not the exact date. After excluding individuals who died within 1 year of registration in the NDRS (*n* = 763 and *n* = 10, respectively), had a previous diagnosis of kidney failure (*n* = 193 and *n* = 9, respectively), and applying a 1-year lag after the index date, considering the latency period of kidney failure (*n* = 1,111 and *n* = 16, respectively), 155,754 individuals with ID and 22,388 individuals with ASD were included in the analysis. Individuals without ID or ASD were selected from the NHIS database as the control group, using a 1:3 matching by age, sex, and index date. The final ID cohort included 155,729 individuals with ID, and 467,187 matched controls without ID. The final ASD cohort included 22,385 individuals with ASD and 67,155 matched controls without ASD, as described in Supplementary Fig. 1 (Online Resource 1).

### Definition of ID and ASD

We defined ID and ASD using NDRS data linked to the NHIS database. Registration for ID requires documentation from a psychiatrist, neurologist, or rehabilitation medicine physician, with intelligence quotient test scores of < 70 [11].

Registration for ASD requires documentation from a child and adolescent psychiatrist, based on the diagnostic criteria for ASD according to the 10th International Classification of Diseases (ICD-10) [11].

### Study outcomes

The study outcome was kidney failure, identified using special V-codes indicating registration for kidney replacement therapy: hemodialysis, V001; peritoneal dialysis, V003; and kidney transplantation, V005 [13]. In Korea, patients with kidney failure registered with these V-codes qualify for the Medical Aid Program, which fully covers dialysis-related medical expenses. Hence, the diagnostic coding for kidney failure is strictly regulated, thereby enhancing diagnostic accuracy. The study population was followed up from the index date to December 31, 2023.

### Covariates

The demographic characteristics (age, sex, income level, and residential area) were retrieved from the NHIS eligibility database. The Charlson Comorbidity Index (CCI) [14] and specified comorbidities were defined using diagnostic codes from the NHIS claims data. Relevant ICD-10 codes with medication prescription records were used to identify diabetes mellitus (E11–E14 with antidiabetic medications), hypertension (ICD-10 codes I10–I13, I15 with antihypertensive medications), dyslipidemia (ICD-10 code E78 with antihyperlipidemic medications), and neuropsychiatric disorders, including mood disorders (depressive disorder and bipolar disorder, F30–F33), schizophrenia (F20), anxiety (F40–F41), and epilepsy (G40–G41, R56, F80.3).

### Statistical analysis

The baseline characteristics of study participants were compared using the t-test for continuous variables and the chi-square test for categorical variables. Kaplan–Meier plots were used to illustrate the cumulative incidence probability of kidney failure in individuals with ID compared with those without ID, and in individuals with ASD compared with those without ASD. Cox proportional hazard analyses were used to calculate hazard ratios (HRs) and 95% confidence intervals (CIs) for kidney failure risk. The proportional hazard assumption was examined using Schonfeld’s residuals. We adjusted for covariates using these two models. Model 1 was adjusted for age, sex, income level, residential area, and Charlson Comorbidity Index score. Model 2 was adjusted for the covariates in Model 1 plus hypertension, diabetes mellitus, dyslipidemia, and neuropsychiatric disorders. Furthermore, we performed subgroup analyses of kidney failure risk among individuals with ID and ASD by sex, age group (< 20 years vs. ≥ 20 years), income level, residential area, and comorbidities, including diabetes, hypertension, dyslipidemia, and neuropsychiatric disorders.

Data analyses were performed using SAS, version 9.4 (SAS Institute Inc., Cary, NC, USA). Statistical significance was defined as a two-sided *p-value* < 0.05.

## Results

### Baseline characteristics

Among individuals with ID, 60.9% were males, and the mean (standard deviation [SD]) age was 23.6 (17.4) years (Table 1). Individuals with ID were more likely than those without ID to receive medical aid, belong to lower-income groups, reside in rural areas, have higher CCI scores, and present with comorbidities, including diabetes, hypertension, dyslipidemia, and psychiatric disorders.

**Table 1:** Baseline characteristics of the study population

	ID			ASD		
	Non-ID control	ID	*p* value	Non-ASD control	ASD	*p* value
	(*n* = 467,187)	(*n* = 155,729)		(*n* = 67,155)	(*n* = 22,385)	
Sex			1			1
Male	284,295 (60.9)	94,765 (60.9)		55,647 (82.9)	18,549 (82.9)	
Female	182,892 (39.1)	60,964 (39.1)		11,508 (17.1)	3,836 (17.1)	
Age, years	23.6 ± 17.4	23.6 ± 17.4	1	7.8 ± 5.3	7.8 ± 5.3	1
Age group, years			1			1
< 20	258,216 (55.3)	86,072 (55.3)		65,091 (96.9)	21,697 (96.9)	
≥ 20	208,971 (44.7)	69,657 (44.7)		2,064 (3.1)	688 (3.1)	
Income			< 0.001			< 0.001
Medical aid	11,613 (2.5)	38,590 (24.8)		1,520 (2.3)	959 (4.3)	
Quartile 1	72,963 (15.6)	27,998 (18.0)		8,116 (12.1)	2,405 (10.7)	
Quartile 2	88,623 (19.0)	27,442 (17.6)		11,005 (16.4)	3,617 (16.2)	
Quartile 3	126,763 (27.1)	31,661 (20.3)		21,123 (31.5)	6,481 (29.0)	
Quartile 4	167,225 (35.8)	30,038 (19.3)		25,391 (37.8)	8,923 (39.9)	
Residential area			< 0.001			< 0.001
Metropolitan	205,835 (44.1)	55,628 (35.7)		29,591 (44.1)	10,333 (46.2)	
Urban	122,841 (26.3)	37,060 (23.8)		17,634 (26.3)	5,776 (25.8)	
Rural	138,511 (29.7)	63,041 (40.5)		19,930 (29.7)	6,276 (28.0)	
CCI categories			< 0.001			< 0.001
0	316,470 (67.7)	95,389 (61.3)		40,296 (60.0)	14,023 (62.6)	
1	108,473 (23.2)	36,270 (23.3)		24,994 (37.2)	7,473 (33.4)	
≥ 2	42,244 (9.0)	24,070 (15.5)		1,865 (2.8)	889 (4.0)	
Diabetes mellitus	14,650 (3.1)	8,804 (5.7)	< 0.001	215 (0.3)	162 (0.7)	< 0.001
Hypertension	22,760 (4.9)	10,085 (6.5)	< 0.001	109 (0.2)	70 (0.3)	< 0.001
Dyslipidemia	26,092 (5.6)	12,467 (8.0)	< 0.001	671 (1.0)	355 (1.6)	< 0.001
Neuropsychiatric disorder	17,662 (3.8)	49,433 (31.7)	< 0.001	1,096 (1.6)	4,673 (20.9)	< 0.001
Mood disorder	6,645 (1.4)	21,754 (14.0)	< 0.001	186 (0.3)	2,342 (10.5)	< 0.001
Schizophrenia	576 (0.1)	9,725 (6.2)	< 0.001	17 (0.03)	675 (3.0)	< 0.001
Anxiety	10,533 (2.3)	13,419 (8.6)	< 0.001	239 (0.4)	984 (4.4)	< 0.001
Epilepsy	3,498 (0.8)	23,868 (15.3)	< 0.001	747 (1.1)	1,964 (8.8)	< 0.001

ID, intellectual disability; ASD, autism spectrum disorder; CCI, Charlson Comorbidity Index

Data are presented as numbers (percentage), mean ± standard deviation, or median (interquartile range)

Meanwhile, the ASD population predominantly comprised males (82.9%) and was younger, with a mean (SD) age of 7.8 (5.3) years. Individuals with ASD were more likely than those without ASD to receive medical aid, reside in metropolitan areas, and present with comorbidities, including diabetes, hypertension, dyslipidemia, and psychiatric disorders.

### Kidney failure risk in individuals with ID and ASD

**Fig. 1:**
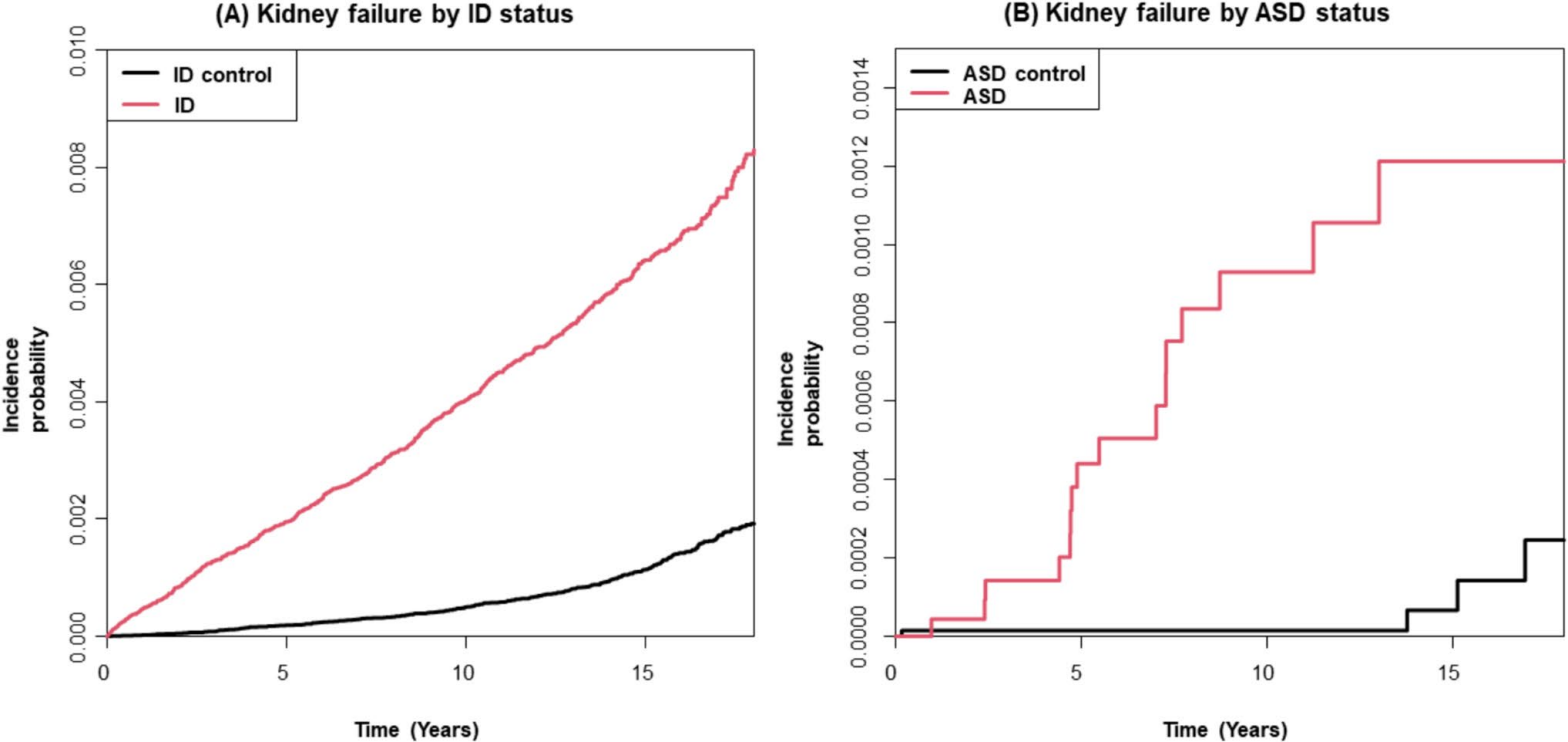
Kaplan–Meier curves illustrating the incidence probability of kidney failure in (**A**) individuals with intellectual disability (ID) compared with those without ID, and (**B**) individuals with autism spectrum disorder (ASD) compared with those without ASD

During a mean follow-up of 10.7 years (SD 5.2), 1,058 kidney failure events occurred in the ID cohort: 687 among individuals with ID and 371 among those without ID (Table 2). For the ASD cohort, during a mean follow-up of 9.3 years (SD 5.3), 20 kidney failure events were identified: 16 among individuals with ASD and 4 among individuals without kidney failure. The cumulative incidence probabilities of kidney failure in the ID and ASD cohorts are shown in Fig. 1.

**Table 2:** Association of intellectual disability (ID) and autism spectrum disorder (ASD) with risk of kidney failure

					HR (95% CI)		
	N	Kidney failure (n)	Duration (PY)	IR (per 1000 PY)	Crude model	Model 1	Model 2
Non-ID	467,187	371	5,057,698.9	0.07	1 (Ref.)	1 (Ref.)	1 (Ref.)
ID	155,729	687	1,620,742.0	0.42	5.83 (5.14‒6.62)	5.12 (4.46‒5.89)	5.50 (4.75‒6.36)
Non-ASD	67,155	4	622,975.0	0.01	1 (Ref.)	1 (Ref.)	1 (Ref.)
ASD	22,385	16	206,446.3	0.08	12.08 (4.04‒36.14)	10.06 (3.32‒30.52)	8.62 (2.68‒27.74)

N, number; IR, incidence rate; PY, person-years; HR, hazard ratio; CI, confidence interval

Model 1: adjusted for age, sex, income level, residential area, and Charlson Comorbidity Index

Model 2: adjusted for age, sex, income level, residential area, Charlson Comorbidity Index, hypertension, diabetes mellitus, dyslipidemia, and neuropsychiatric disorders

Individuals with ID had a higher risk of developing kidney failure than those without ID: HR 5.83 (95% CI 5.14–6.62), and this elevated risk persisted after full adjustment for covariates: adjusted HR (aHR) 5.50 (95% CI 4.75–6.36).

Individuals with ASD showed an even higher HR of 12.08 (95% CI 4.04–36.14) than those without ASD. After full adjustment for covariates, the risk was attenuated but remained elevated for individuals with ASD: aHR 8.62 (95% CI 2.68–27.74).

### Subgroup analyses in individuals with ID and ASD

The association between ID and kidney failure across demographic characteristics and comorbidities is shown in Supplementary Table 1 (Online Resource 2). The increased risk of kidney failure associated with ID was more pronounced among females (aHR 6.80, 95% CI 4.03–5.73) than males (aHR 4.80, 95% CI 5.46–8.48, *p* for interaction 0.009), and among those aged < 20 years (aHR 17.88, 95% CI 11.88–26.89) compared with those aged ≥ 20 years old (aHR 4.31, 95% CI 3.67–5.05, *p* for interaction < 0.001). Individuals in the higher income levels showed a more pronounced risk for kidney failure (quartile 4, aHR 6.95, 95% CI 5.29–9.12 vs. medical aid, aHR 2.22, 95% CI 1.49–3.32, p for interaction < 0.001). Those without comorbidities also had a higher risk of kidney failure than those with comorbidities, including diabetes mellitus (*p* < 0.001), hypertension (*p* < 0.001), dyslipidemia (*p* = 0.003), and neuropsychiatric disorders (*p* = 0.009).

Meanwhile, no significant interactions were observed between ASD and demographic characteristics or comorbidities for kidney failure risk (Supplementary Table 2 in Online Resource 2).

## Discussion

In this population-based nationwide study, individuals with ID or ASD had an elevated risk of incident kidney failure compared with non-ID or non-ASD controls. In subgroup analyses, ID and ASD were associated with an increased risk of kidney failure in most subgroups.

In our analyses, individuals with developmental disabilities had an elevated risk of developing kidney failure than controls, with aHRs of 5.50 for ID and 8.62 for ASD. Evidence on incident CKD or kidney failure risk in individuals with developmental disabilities remains scarce; only one study investigated the incidence of advanced CKD, defined as CKD at stage 4 or later, in individuals with developmental disabilities [15]. A previous study reported that developmental disabilities were associated with a two-fold increase in the incidence of advanced CKD after adjusting for demographics, hypertension, and diabetes, demonstrating a weaker association than that observed in our study. However, that study was performed using non-representative samples from a single private payer database, included a much smaller number of individuals with developmental disabilities (*n* = 33,561), followed them for a shorter period (≤ 4 years) than our study, and was limited to adults. Our large-scale study based on a complete disability registry incorporating all individuals registered with ID or ASD in Korea revealed a strong association between ID/ASD and kidney failure.

Examining baseline characteristics of the study population revealed that individuals with ID or ASD were more likely to be in the lowest income level, have a high CCI score (≥ 2), and have more comorbidities compared with age- and sex- matched controls, consistent with previous studies [3, 4, 16]. However, although the association between ID/ASD and kidney failure was attenuated after adjusting for these covariates, it remained highly significant. This finding implies that factors other than comorbidities, including diabetes and hypertension or poor economic status, may also substantially contribute to the elevated kidney failure risk in individuals with ID/ASD.

One possible mediator between ID/ASD and incident kidney failure is obesity, which is an independent risk factor for kidney failure [6], and studies have suggested that ID/ASD is associated with obesity [4, 17]. Several factors, including unhealthy lifestyles, frequent use of psychotropic medication, and genetic syndromes associated with ID/ASD, may, at least partly, contribute to obesity and a subsequent increase in the risk of kidney failure among this population [4, 17]. Individuals with ID/ASD may also face challenges in practicing healthy habits, such as regular physical activity [18], which are linked to kidney function [19]. In addition, disparities in healthcare utilization are common among individuals with developmental disabilities [18]. Populations with developmental disabilities may experience limited access and social barriers to health screening or preventive services, resulting in an increased risk of disease progression and poor health outcomes, including kidney failure [18, 20]. Additionally, specific genetic syndromes or genetic variants that are associated with both developmental disabilities and certain kidney diseases may partly contribute to an elevated kidney failure risk [21, 22]. In the current study, the retrieved dataset did not include information on potential contributors to kidney failure risk. Future investigations should investigate disparities in various factors, including obesity, health habits, and healthcare accessibility, between individuals with and without ID/ASD, and their impact on differences in kidney failure risk.

Subgroup analyses revealed that ID and ASD were associated with an elevated risk of kidney failure in most subgroups, whereas age, sex, income level, and comorbidities demonstrated statistically significant interactions with ID. The magnitude of the association between ID and kidney failure was greater in females than in males. Factors such as obesity, unhealthy lifestyle, and low motivation for health promotion are reportedly less prevalent in females than in males [23, 24], and these risk factors may be more strongly affected by the presence of ID, which could explain the more pronounced effect of ID on kidney failure risk in females. Additionally, barriers to medical care, such as discrimination from healthcare providers, may differ according to the patient [25]. The elevated risk of kidney failure according to ID was more pronounced in the younger age group (< 20 years) than in the older age group (aged ≥ 20 years), which is consistent with the results of a previous study [15]. Because various risk factors for kidney failure, such as obesity and metabolic disorders, are more prevalent in adults than in children [23], the differences in these factors according to ID may be smaller in older age groups. Also, the risk increase by specific genetic variants linked to both congenital kidney anomalies and ID may appear more pronounced at younger ages, as kidney failure attributable to congenital anomalies is more likely to occur relatively early in life [22]. Among individuals with ID, those with higher income levels showed a more pronounced risk for kidney failure. Individuals with a higher economic status are typically more likely to receive better medical services and have sufficient access to healthcare [26]; therefore, differences in these factors by ID may be more apparent in higher income groups than in lower-income groups. The association between ID and kidney failure risk was also more prominent among the subgroups without comorbidities than among those with comorbidities. These comorbidities may be associated with obesity or unhealthy habits such as physical inactivity [27, 28], contributing to the attenuated effect of ID on kidney failure risk. Our findings imply that in populations generally considered to be at a lower risk of kidney failure—and thus likely to receive less clinical attention, such as females, children, individuals with high socioeconomic status, and those without comorbidities [6]—the increase in kidney failure risk associated with ID may be more pronounced. Surveillance and prevention strategies for kidney failure are warranted in populations affected by ID. Among individuals with ASD, no significant interactions with demographic characteristics or comorbidities were observed with regard to the risk of kidney failure, possibly due to the insufficient statistical power from the small number of events.

Individuals with ID or ASD experience a high burden of comorbidities and premature mortality [3, 4, 8, 9], accompanied by a poor economic status and a high unemployment rate [16]. The increased incidence of kidney failure observed in our study may further exacerbate their burden of disease, mortality, and healthcare expenditure, posing a serious concern [1, 2]. Despite the high potential risk of morbidity and consequent medical costs, utilization of preventive health services among this population appears to be low, which may further accelerate the long-term increase in disease burden [18]. Our study implies that systematic efforts by the government and public health professionals are required to promote more proactive surveillance for detecting CKD at an earlier stage and preventing kidney failure among populations with ID or ASD.

The strengths of our study include its large population-based dataset based on complete nationwide registers of disability. Using this dataset, we identified all individuals registered with ID or ASD in Korea and followed them for a prolonged period (approximately 10 years), thereby providing sufficient statistical power to rigorously assess kidney failure risk. To the best of our knowledge, among the very few longitudinal studies investigating CKD or kidney failure risk among individuals with developmental disabilities, ours is the first population-based nationwide study and includes the largest number of cases of developmental disabilities. In addition, our large-scale dataset allowed for detailed subgroup analyses across various subgroups.

Nevertheless, our study had some limitations. First, as this was a retrospective study using a pre-established database, we lacked information regarding certain factors that may be associated with ID/ASD and kidney failure, such as obesity status and lifestyle. Additional studies are warranted to determine potential mediators of ID/ASD and kidney failure. Second, a marginal proportion of individuals with ID/ASD may not have been captured in the NDRS and therefore were not included in this study. However, such cases are likely to represent milder disease severity; accordingly, the present study has relevance in that it focuses on individuals with more clearly defined ID/ASD. In addition, individuals with overlapping disabilities may, in some instances, have been classified under other disability categories and thus were not included in the study. Third, patients with kidney failure who did not receive kidney replacement therapy and therefore lacked records of the relevant registration codes may have been missed; however, given the substantial benefits of the registration for copayment reduction program, such cases are presumed to be very rare. Finally, despite the extensive size of the dataset, the number of kidney failure events was relatively small in the ASD cohort. The relatively young age of the ASD cohort may have partly contributed to the small number of events. Future investigations, including a larger population and a longer follow-up period, may be required to adequately assess kidney failure risk in patients with ASD.

In conclusion, the risk of incident kidney failure was higher in individuals with ID or ASD than in the controls. Public health strategies aimed at improving surveillance and prevention of kidney failure are warranted in these populations.

## Supplementary Information

Below is the link to the electronic supplementary material.

Graphical abstract (PPTX 133 KB)

Supplementary file1 (DOCX 79 KB)

Supplementary file2 (DOCX 42 KB)

## Data Availability

The datasets generated during and/or analysed during the current study are not publicly available because they were used under a license for this investigation from NHIS, but are available from the corresponding author on reasonable request with permission from the NHIS.
