# Supplementary material for: Association between intellectual disability and autism spectrum disorder with kidney failure
Source: Pediatr Nephrol. 2026 Feb 1;41(7):2063–70. doi: 10.1007/s00467-026-07177-x (PMC13197328; doi:10.1007/s00467-026-07177-x)
Supplement: Supplementary file 1 — Graphical abstract (PPTX 133 KB) [file 467_2026_7177_MOESM1_ESM.pptx]

## Slide 1
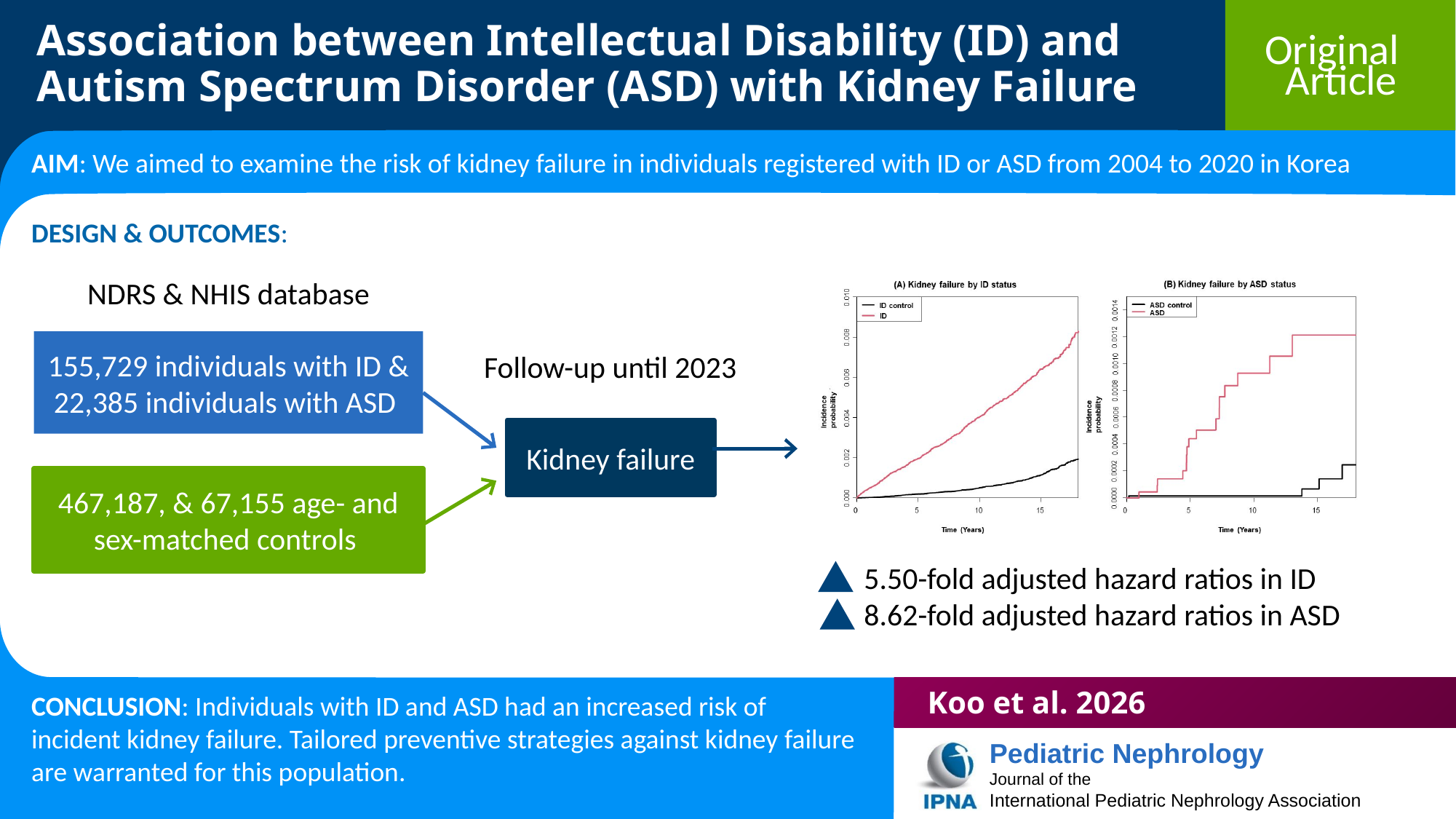

Association between Intellectual Disability (ID) and Autism Spectrum Disorder (ASD) with Kidney Failure
AIM: We aimed to examine the risk of kidney failure in individuals registered with ID or ASD from 2004 to 2020 in Korea
DESIGN & OUTCOMES:
NDRS & NHIS database
155,729 individuals with ID & 22,385 individuals with ASD
Follow-up until 2023
Kidney failure
467,187, & 67,155 age- and sex-matched controls
5.50-fold adjusted hazard ratios in ID
8.62-fold adjusted hazard ratios in ASD
Koo et al. 2026
CONCLUSION: Individuals with ID and ASD had an increased risk of incident kidney failure. Tailored preventive strategies against kidney failure are warranted for this population.
