# Supplementary material for: Association between intellectual disability and autism spectrum disorder with kidney failure
Source: Pediatr Nephrol. 2026 Feb 1;41(7):2063–70. doi: 10.1007/s00467-026-07177-x (PMC13197328; doi:10.1007/s00467-026-07177-x)
Supplement: Supplementary file 2 — Supplementary file1 (DOCX 79 KB) [file 467_2026_7177_MOESM2_ESM.docx]

**Article title**: Association between Intellectual Disability and Autism Spectrum Disorder with Kidney Failure

**Journal name**: Pediatric nephrology

**Author names**: Hye Yeon Koo, MD, MPH; In Young Cho, MD, MPH^a^; Yong-Moon Mark Park, MD, PhD; Kyung Mee Kim, PhD; Chung Eun Lee, PhD; Kyungdo Han, PhD^b^

^a^ Department of Family Medicine and Supportive Care Center, Samsung Medical Center, Sungkyunkwan University School of Medicine, Seoul, Republic of Korea; E-mail: ciyoung0604@gmail.com

^b^ Department of Statistics and Actuarial Science, Soongsil University, Seoul, Republic of Korea; E-mail: hkd917@naver.com

**Supplementary Figure**

**
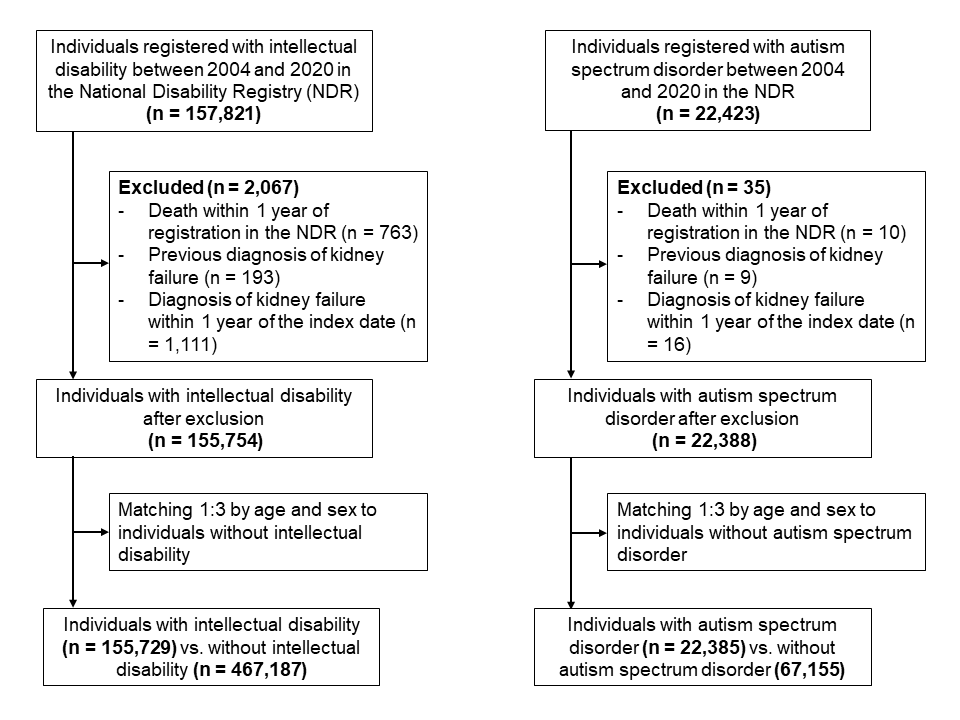
**

**Supplementary Fig. 1** Flowchart of the study population inclusion process.
