# Supplementary material for: Association between intellectual disability and autism spectrum disorder with kidney failure
Source: Pediatr Nephrol. 2026 Feb 1;41(7):2063–70. doi: 10.1007/s00467-026-07177-x (PMC13197328; doi:10.1007/s00467-026-07177-x)
Supplement: Supplementary file 3 — Supplementary file2 (DOCX 42 KB) [file 467_2026_7177_MOESM3_ESM.docx]

**Article title**: Association between Intellectual Disability and Autism Spectrum Disorder with Kidney Failure

**Journal name**: Pediatric nephrology

**Author names**: Hye Yeon Koo, MD, MPH; In Young Cho, MD, MPH^a^; Yong-Moon Mark Park, MD, PhD; Kyung Mee Kim, PhD; Chung Eun Lee, PhD; Kyungdo Han, PhD^b^

^a^ Department of Family Medicine and Supportive Care Center, Samsung Medical Center, Sungkyunkwan University School of Medicine, Seoul, Republic of Korea; E-mail: ciyoung0604@gmail.com

^b^ Department of Statistics and Actuarial Science, Soongsil University, Seoul, Republic of Korea; E-mail: hkd917@naver.com

**Supplementary Tables**

**Supplementary Table 1** Association between intellectual disability (ID) and the risk of kidney failure across various subgroups

| Subgroups | ID Status | N | IR (per 1000 PY) | Crude HR (95% CI) | *p* for interaction | Adjusted HR^*^ (95% CI) | *p* for interaction |
| --- | --- | --- | --- | --- | --- | --- | --- |
| Sex |  |  |  |  |  |  |  |
| Male | Non-ID | 284,295 | 0.08 | 1 (Ref.) | 0.002 | 1 (Ref.) | 0.009 |
|  | ID | 94,765 | 0.39 | 4.95 (4.22‒5.81) |  | 4.80 (4.03‒5.73) |  |
| Female | Non-ID | 182,892 | 0.06 | 1 (Ref.) |  | 1 (Ref.) |  |
|  | ID | 60,964 | 0.48 | 7.52 (6.12‒9.25) |  | 6.80 (5.46‒8.48) |  |
| Age groups |  |  |  |  |  |  |  |
| < 20 years | Non-ID | 258,216 | 0.01 | 1 (Ref.) | <0.001 | 1 (Ref.) | <0.001 |
|  | ID | 86,072 | 0.20 | 19.50 (13.00‒29.25) |  | 17.88 (11.88‒26.89) |  |
| ≥ 20 years | Non-ID | 208,971 | 0.14 | 1 (Ref.) |  | 1 (Ref.) |  |
|  | ID | 69,657 | 0.68 | 4.86 (4.24‒5.58) |  | 4.31 (3.67‒5.05) |  |
| Income level |  |  |  |  |  |  |  |
| Medical aid | Non-ID | 11,613 | 0.21 | 1 (Ref.) | 0.007 | 1 (Ref.) | <0.001 |
|  | ID | 38,590 | 0.53 | 2.51 (1.68‒3.74) |  | 2.22 (1.49‒3.32) |  |
| Quartile 1 | Non-ID | 72,963 | 0.09 | 1 (Ref.) |  | 1 (Ref.) |  |
|  | ID | 27,998 | 0.46 | 5.12 (3.83‒6.84) |  | 5.35 (3.99‒7.17) |  |
| Quartile 2 | Non-ID | 88,623 | 0.08 | 1 (Ref.) |  | 1 (Ref.) |  |
|  | ID | 27,442 | 0.37 | 4.86 (3.62‒6.52) |  | 5.10 (3.78‒6.87) |  |
| Quartile 3 | Non-ID | 126,763 | 0.06 | 1 (Ref.) |  | 1 (Ref.) |  |
|  | ID | 31,661 | 0.36 | 5.79 (4.40‒7.62) |  | 6.47 (4.90‒8.56) |  |
| Quartile 4 | Non-ID | 167,225 | 0.06 | 1 (Ref.) |  | 1 (Ref.) |  |
|  | ID | 30,038 | 0.36 | 5.87 (4.50‒7.66) |  | 6.95 (5.29‒9.12) |  |
| Residential area |  |  |  |  |  |  |  |
| Metropolitan | Non-ID | 205,835 | 0.07 | 1 (Ref.) | 0.536 | 1 (Ref.) | 0.169 |
|  | ID | 55,628 | 0.39 | 5.60 (4.57‒6.86) |  | 5.55 (4.48‒6.88) |  |
| Urban | Non-ID | 122,841 | 0.07 | 1 (Ref.) |  | 1 (Ref.) |  |
|  | ID | 37,060 | 0.46 | 6.54 (5.09‒8.39) |  | 6.51 (5.03‒8.43) |  |
| Rural | Non-ID | 138,511 | 0.08 | 1 (Ref.) |  | 1 (Ref.) |  |
|  | ID | 63,041 | 0.43 | 5.49 (4.43‒6.80) |  | 4.75 (3.78‒5.96) |  |
| Diabetes mellitus |  |  |  |  |  |  |  |
| No | Non-ID | 452,537 | 0.04 | 1 (Ref.) | <0.001 | 1 (Ref.) | <0.001 |
|  | ID | 146,925 | 0.30 | 6.91 (5.89‒8.12) |  | 6.82 (5.74‒8.12) |  |
| Yes | Non-ID | 14,650 | 1.08 | 1 (Ref.) |  | 1 (Ref.) |  |
|  | ID | 8,804 | 3.08 | 3.01 (2.45‒3.71) |  | 3.53 (2.81‒4.43) |  |
| Hypertension |  |  |  |  |  |  |  |
| No | Non-ID | 444,427 | 0.04 | 1(Ref.) | <0.001 | 1 (Ref.) | <0.001 |
|  | ID | 145,644 | 0.29 | 7.54 (6.36‒8.94) |  | 7.14 (5.95‒8.56) |  |
| Yes | Non-ID | 22,760 | 0.78 | 1 (Ref.) |  | 1 (Ref.) |  |
|  | ID | 10,085 | 2.83 | 3.84 (3.16‒4.66) |  | 3.59 (2.89‒4.46) |  |
| Dyslipidemia |  |  |  |  |  |  |  |
| No | Non-ID | 441,095 | 0.05 | 1 (Ref.) | <0.001 | 1 (Ref.) | 0.003 |
|  | ID | 143,262 | 0.33 | 6.61 (5.67‒7.71) |  | 6.17 (5.22‒7.29) |  |
| Yes | Non-ID | 26,092 | 0.56 | 1 (Ref.) |  | 1 (Ref.) |  |
|  | ID | 12,467 | 2.05 | 3.93 (3.14‒4.91) |  | 4.07 (3.19‒5.20) |  |
| Neuropsychiatric disorders |  |  |  |  |  |  |  |
| No | Non-ID | 449,525 | 0.07 | 1 (Ref.) | <0.001 | 1 (Ref.) | 0.009 |
|  | ID | 106,296 | 0.38 | 5.68 (4.92‒6.56) |  | 5.90 (5.05‒6.89) |  |
| Yes | Non-ID | 17,662 | 0.26 | 1 (Ref.) |  | 1 (Ref.) |  |
|  | ID | 49,433 | 0.53 | 2.10 (1.53‒2.88) |  | 3.70 (2.68‒5.11) |  |

Abbreviations: N, number; IR, incidence rate; PY, person-years; HR, hazard ratio; CI, confidence interval.

^*^Adjusted for age, sex, income level, residential area, Charlson Comorbidity Index, hypertension, diabetes mellitus, dyslipidemia, and neuropsychiatric disorders.

**Supplementary Table 2** Association between autism spectrum disorder (ASD) and the risk of kidney failure across various subgroups

| Subgroups | ASD | N | IR (per 1000 PY) | Crude HR (95% CI) | *p* for interaction | Adjusted HR^*^ (95% CI) | *p* for interaction |
| --- | --- | --- | --- | --- | --- | --- | --- |
| Sex |  |  |  |  |  |  |  |
| Male | Non-ASD | 55,647 | 0.01 | 1 (Ref.) | 0.804 | 1 (Ref.) | 0.731 |
|  | ASD | 18,549 | 0.06 | 11.06 (3.09‒39.66) |  | 7.61 (2.01‒28.88) |  |
| Female | Non-ASD | 11,508 | 0.01 | 1 (Ref.) |  | 1 (Ref.) |  |
|  | ASD | 3,836 | 0.14 | 15.17 (1.77‒129.84) |  | 12.08 (1.21‒120.81) |  |
| Age groups |  |  |  |  |  |  |  |
| <20 years | Non-ASD | 65,091 | 0.01 | 1 (Ref.) | N/A | 1 (Ref.) | N/A |
|  | ASD | 21,697 | 0.08 | 12.08 (4.04‒36.12) |  | 8.87 (2.73‒28.87) |  |
| ≥20 years | Non-ASD | 2,064 | 0.00 | 1 (Ref.) |  | 1 (Ref.) |  |
|  | ASD | 688 | 0.00 | N/A |  | N/A |  |
| Income levels |  |  |  |  |  |  |  |
| Medical aid | Non-ASD | 1,520 | 0.06 | 1 (Ref.) | 0.984 | 1 (Ref.) | 0.952 |
|  | ASD | 959 | 0.10 | 1.58 (0.10‒25.23) |  | 0.81 (0.05‒13.48) |  |
| Quartile 1 | Non-ASD | 8,116 | 0.00 | 1 (Ref.) |  | 1 (Ref.) |  |
|  | ASD | 2,405 | 0.05 | N/A |  | N/A |  |
| Quartile 2 | Non-ASD | 11,005 | 0.03 | 1 (Ref.) |  | 1 (Ref.) |  |
|  | ASD | 3,617 | 0.11 | 4.26 (0.95‒19.02) |  | 3.12 (0.64‒15.16) |  |
| Quartile 3 | Non-ASD | 21,123 | 0.00 | 1 (Ref.) |  | 1 (Ref.) |  |
|  | ASD | 6,481 | 0.12 | N/A |  | N/A |  |
| Quartile 4 | Non-ASD | 25,391 | 0.00 | 1 (Ref.) |  | 1 (Ref.) |  |
|  | ASD | 8,923 | 0.04 | N/A |  | N/A |  |
| Residential area |  |  |  |  |  |  |  |
| Metropolitan | Non-ASD | 29,591 | 0.00 | 1 (Ref.) | 0.908 | 1(Ref.) | 0.893 |
|  | ASD | 10,333 | 0.06 | 17.59 (2.12‒146.12) |  | 11.40 (1.32‒98.83) |  |
| Urban | Non-ASD | 17,634 | 0.01 | 1 (Ref.) |  | 1 (Ref.) |  |
|  | ASD | 5,776 | 0.13 | 10.29 (2.14‒49.53) |  | 6.43 (1.27‒32.53) |  |
| Rural | Non-ASD | 19,930 | 0.01 | 1 (Ref.) |  | 1 (Ref.) |  |
|  | ASD | 6,276 | 0.05 | 9.73 (1.01‒93.52) |  | 10.64 (0.96‒118.70) |  |
| Diabetes mellitus |  |  |  |  |  |  |  |
| No | Non-ASD | 66,940 | 0.01 | 1 (Ref.) | N/A | 1 (Ref.) | N/A |
|  | ASD | 22,223 | 0.07 | 11.38 (3.78‒34.28) |  | 8.40 (2.59‒27.17) |  |
| Yes | Non-ASD | 215 | 0.00 | 1 (Ref.) |  | 1 (Ref.) |  |
|  | ASD | 162 | 0.70 | N/A |  | N/A |  |
| Hypertension |  |  |  |  |  |  |  |
| No | Non-ASD | 67,046 | 0.01 | 1 (Ref.) | N/A | 1 (Ref.) | N/A |
|  | ASD | 22,315 | 0.08 | 12.10 (4.05‒36.20) |  | 8.62 (2.68‒27.74) |  |
| Yes | Non-ASD | 109 | 0.00 | 1 (Ref.) |  | 1 (Ref.) |  |
|  | ASD | 70 | 0.00 | N/A |  | N/A |  |
| Dyslipidemia |  |  |  |  |  |  |  |
| No | Non-ASD | 66,484 | 0.01 | 1 (Ref.) | N/A | 1 (Ref.) | N/A |
|  | ASD | 22,030 | 0.07 | 11.39 (3.78‒34.31) |  | 8.08 (2.48‒26.31) |  |
| Yes | Non-ASD | 671 | 0.00 | 1 (Ref.) |  | 1 (Ref.) |  |
|  | ASD | 355 | 0.40 | N/A |  | N/A |  |
| Neuropsychiatric disorder |  |  |  |  |  |  |  |
| No | Non-ASD | 66,059 | 0.01 | 1 (Ref.) | 0.989 | 1 (Ref.) | 0.992 |
|  | ASD | 17,712 | 0.06 | 8.49 (2.61‒27.55) |  | 7.93 (2.40‒26.19) |  |
| Yes | Non-ASD | 1,096 | 0.00 | 1 (Ref.) |  | 1 (Ref.) |  |
|  | ASD | 4,673 | 0.16 | N/A |  | N/A |  |

Abbreviations: N, number; IR, incidence rate; PY, person-years; HR, hazard ratio; CI, confidence interval; N/A, not available.

N/A indicates that the estimates were not calculated because of a lack of events in the subgroup.

^*^Adjusted for age, sex, income level, residential area, Charlson Comorbidity Index, hypertension, diabetes mellitus, dyslipidemia, and neuropsychiatric disorders.
